# Mathematical models of cancer metabolism

**DOI:** 10.1186/s40170-015-0140-6

**Published:** 2015-12-22

**Authors:** Elke Katrin Markert, Alexei Vazquez

**Affiliations:** Institute of Cancer Sciences, University of Glasgow, Garscube Estate, Switchback Road, Glasgow, G61 1BD UK; Cancer Research UK Beatson Institute, Garscube Estate, Switchback Road, Glasgow, G61 1BD UK

## Abstract

Metabolism is essential for life, and its alteration is implicated in multiple human diseases. The transformation from a normal to a cancerous cell requires metabolic changes to fuel the high metabolic demands of cancer cells, including but not limited to cell proliferation and cell migration. In recent years, there have been a number of new discoveries connecting known aberrations in oncogenic and tumour suppressor pathways with metabolic alterations required to sustain cell proliferation and migration. However, an understanding of the selective advantage of these metabolic alterations is still lacking. Here, we review the literature on mathematical models of metabolism, with an emphasis on their contribution to the identification of the selective advantage of metabolic phenotypes that seem otherwise wasteful or accidental. We will show how the molecular hallmarks of cancer can be related to cell proliferation and tissue remodelling, the two major physiological requirements for the development of a multicellular structure. We will cover different areas such as genome-wide gene expression analysis, flux balance models, kinetic models, reaction diffusion models and models of the tumour microenvironment. We will also highlight current challenges and how their resolution will help to achieve a better understanding of cancer metabolism and the metabolic vulnerabilities of cancers.

## Background

The hallmarks of cancer [1] highlight major processes and mechanisms required for cancer development. They are divided into *core hallmarks*, major phenotypes needed to form a cancer, and the *enabling hallmark* of genomic instability, a molecular mechanism driving the acquisition of the core hallmarks. In the most recent update to this system [2], metabolism is described as an *emerging hallmark* because metabolism is commonly altered in cancer. The designation “emerging” reflects a sense of ambiguity (neither core nor enabling) concerning the role of metabolism in cancer development. Indeed, the authors note that the metabolic alterations observed in cancer could simply be a consequence of acquisition of the core hallmarks.

**Fig. 1:**
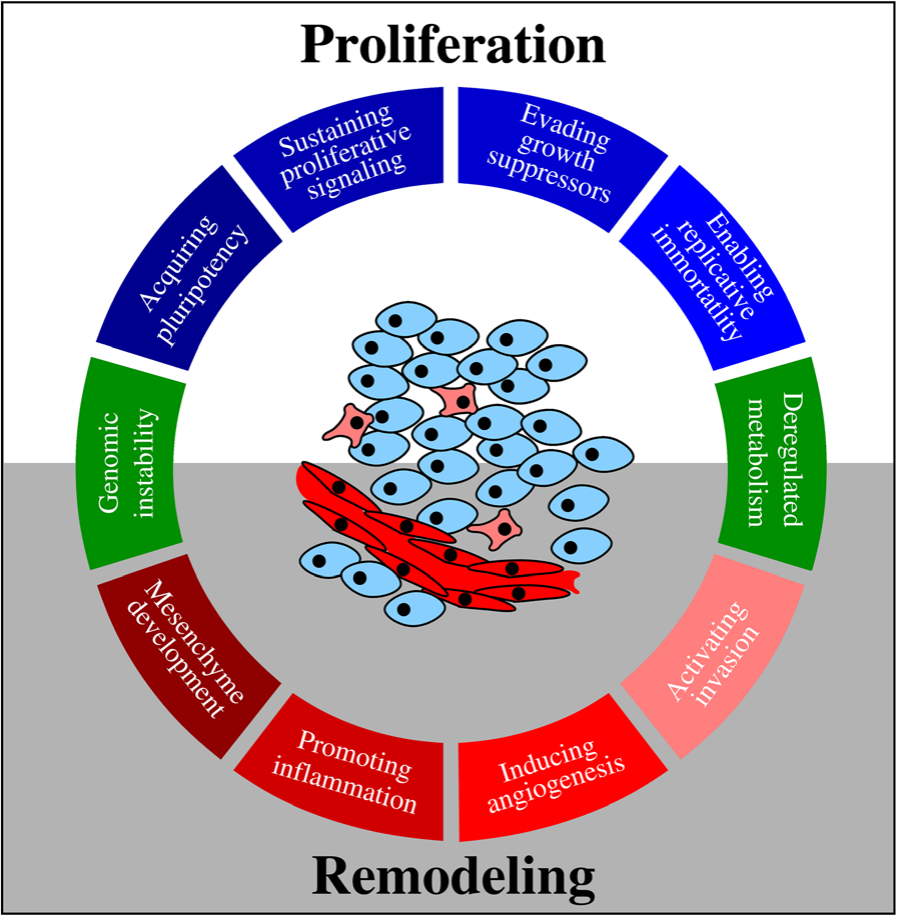
The hallmarks of cancer. The core hallmarks of cancer are arranged around the circle. Given their function, the molecular hallmarks are divided into those promoting cell proliferation (*white background*) and those promoting tissue remodelling (*grey background*). Genome instability has a special location in between because it is the molecular mechanism driving the emergence of the other hallmarks and the same time the potential transition between the physiological states of proliferation and remodelling. Deregulated metabolism has also a special location in between because it is a necessary requirement for both cell proliferation and tissue remodelling, and at the same time, metabolic alterations may be the cause or drive transitions between proliferation and remodelling

The hallmarks describe the aberrations from a normal functioning organism that define cancer as a disease. There is a complementary view based on the physiology of cancer as a *developing tissue* ([3], Fig. 1). In this physiological view, the core hallmarks are interpreted as the molecular pathways necessary to establish two essential requirements for the development of a multicellular structure: cell proliferation and tissue remodelling. In the following, we refer to these as *physiological hallmarks*. In a tumour, cell proliferation is required to expand populations of cells with molecular alterations. Tissue remodelling is required to form a consolidated tumour, bringing nutrient supplies, invading nearby tissues and evading the immune system. Both physiological hallmarks require energy and biosynthetic precursors—albeit in possibly different distributions and total amounts—and therefore, metabolism becomes an *enabling hallmark* in this conceptual framework (Fig. 1). In other words, metabolism is the engine fuelling cell proliferation, tissue development and homeostasis.

Here, we review some attempts to harness this metabolism centric framework into mathematical descriptions and quantifiable metrics. The mathematical models are divided into five major categories based on the techniques used and their focus on cell or tumour metabolism. First, we discuss genome-wide gene expression analysis, as our major tool to investigate the heterogeneity of metabolism across cancers of different types. Second, we focus on flux balance models that aim to understand cell metabolism at a steady state. This is followed by a third section reviewing kinetic models of selected cell metabolic pathways and the path to genome-scale models with kinetics. Fourth, we go over metabolic models that take into account the spatial dimensions of the cell, revealing metabolic phenomena that could be determined by spatial heterogeneity within the cell. Finally, we discuss different tumour microenvironment models, focusing on how metabolic interactions between cancer and stroma cells impact tumour growth, invasion and metastasis.

## Review

### Genome-wide gene expression analysis

Gene expression profiles are a unique resource to understand the differential utilization of metabolic pathways across cancers and genome-wide. As of March 2015, expression profiles from about 300,000 samples were deposited in the Gene Expression Omnibus (GEO) database. In spite of a number of caveats, gene expression profiles have the advantage that they have been obtained using a few standard microarrays and that they interrogate gene expression at a genomic level. Additionally, the abundance of data accumulated by now allows researchers to interrogate metabolic expression patterns in a larger context.

Gene expression analysis can be utilized to identify metabolic genes associated with cancer. An investigation of 1981 microarray samples from 19 cancer types identified metabolic genes whose expression is most commonly altered in cancers [4]. Many of the altered metabolic genes have well-known roles in cell proliferation, including one-carbon and nucleotide metabolism, and tissue remodelling, including hypoxia and glycosylation metabolism. Interestingly, the one-carbon metabolism genes *SHMT2* and *MTHFD2*, coding for mitochondrial serine hydromethyltransferase and methylene-tetrahydrofolate dehydrogenase/cyclohydrolase, were identified among the 50 most commonly overexpressed genes. However, the genes *SHMT1* and *MTHFD1* coding for the corresponding cytosolic enzymes were not. This observation resonates with a previous report indicating that high expression of the mitochondrial one-carbon metabolism enzymes correlates with gene signatures of cell proliferation and Myc activation and is predictive of a good response to the antiproliferative agent methotrexate [5].

Gene expression analysis can also be utilized to uncover metabolic subtypes. The gene expression pattern of metabolism genes in a cancer tissue sample should reflect its metabolic state. Although there are many regulatory mechanisms at the post-translational level, there is generally a subset of metabolic genes that is regulated at the transcriptional level. In fact, many transcription factors with relevance to cancer regulate metabolic genes, including Myc [6], HIF1α [7] and p53 [8]. The analysis of the expression patterns of metabolic genes across cancers should therefore uncover major metabolic subtypes. An unsupervised clustering analysis of more than 2500 microarray samples from 22 different tumour types revealed that the metabolic gene expression profiles of tumours are in fact closer to their corresponding normal tissues than to other tumours [9]. This shows that tissue of origin has a major impact on metabolic gene expression profiles even under oncogenic transformation.

Additional information can be obtained after correcting for tissue type. We analysed about 4000 microarray samples from five cancer types that were linked to clinical outcome reports [3]. The signal of tissue type was removed after subtracting the average log_2_ expression of each gene across all samples of each cohort. Working under the hypothesis that cell proliferation and tissue remodelling are the physiological hallmarks characterizing tumour probes on the molecular level (Fig. 1), we investigated the differential expression of gene signatures associated with proliferation and remodelling across cancers. We noted that gene signatures quantifying proliferation exhibit a low but consistently negative correlation with those quantifying remodelling. Next, we performed a supervised clustering of cancers based on their degree of cell proliferation (P) and tissue remodelling (R). This resulted in four distinct subtypes, independently of tissue type: P−/R−, P−/R+, P+/R− and P+/R+. We did not observe significant changes in the percentage or occurrence of either subtype with regard to tissue of origin, suggesting that the physiological hallmarks are features of all (solid) cancers. The P/R subtypes also exhibit distinct survival outcomes. The group with low proliferation-remodelling signatures (P−/R−) has the best outcome independently of the tissue of origin. In contrast, the group with high proliferation-remodelling signatures (P+/R+ group) has the worst outcome, again independently of the tissue of origin. There is however a tissue difference regarding the survival of the intermediate P−/R+ and P+/R− groups. In brain, breast, lung and prostate cancers, the patients in the P+/R− group die earlier than those in the P−/R+ group. In contrast, in colorectal and ovarian cancers, the patients in the P+/R− group die later than those in the P−/R+ group. This shows that the expression of the physiological hallmarks has a severe impact on the most global clinical phenotype of the disease, survival.

The existence of large subsets of tumours expressing predominantly one physiological hallmark may be rooted in metabolic constraints [3]. Highly vascularized tumours may have sufficient nutrient supply to support proliferation, while poorly vascularized ones may devote their limited nutrient resources to remodel the environment to increase the nutrient supply. Indeed, pathways required for cell proliferation, including glycolysis, the pentose phosphate pathway, the TCA cycle, OxPhos, one-carbon metabolism and ribosomes, are positively correlated with the signature for cell proliferation (Table 1). In contrast, a lysosome gene signature is positively correlated with the tissue remodelling signature (Table 1), indicating that autophagy is more active in tumours undergoing tissue remodelling. We also noticed that fatty acid metabolism does not exhibit any specific pattern (Table), indicating that fatty acid metabolism either is a requirement for all proliferative and remodelling subtypes or is associated with a yet unidentified physiological hallmark.

**Table 1:** Association of metabolism with proliferation and remodelling signatures. The Pearson correlation coefficient between the listed gene signatures (rows) and the gene signatures of cell proliferation and tissue remodelling (columns), as obtained from the analysis of about 4000 samples from five cancer types [3]. The genes on the tissue remodelling and cell proliferation and first two groups of signatures were obtained from gene ontology annotations, and they are reported in ref. [3]. The genes in the remaining metabolic signatures were obtained from the KEGG annotations reported in the Molecular Signatures Database (MSigDB) [16]

Signature	Tissue remodelling	Cell proliferation
G1/S transition	−0.07	0.85
DNA replication	−0.12	0.87
Telomere organization	−0.14	0.75
DNA packaging	−0.17	0.81
Chromosome segregation	−0.15	0.81
G2/M transition	−0.15	0.80
Cell division	−0.06	0.87
Cell junction organization	0.66	−0.05
Cell adhesion	0.81	−0.15
Cell migration	0.86	−0.05
Angiogenesis	0.77	−0.04
Cytokine production	0.62	−0.02
Inflammatory response	0.67	−0.07
Response to wounding	0.82	−0.05
Glycolysis	0.08	0.37
Pentose phosphate pathway	0.02	0.37
Oxidative phosphorylation	−0.22	0.34
TCA cycle	−0.12	0.41
Serine, glycine, 1C metabolism	−0.16	0.48
Ribosome	−0.13	0.14
Lysosome	0.38	0.09
Fatty acid metabolism	−0.02	−0.02

Cancer cell lines grown in vitro for several passages retain the antagonism between cell proliferation and tissue remodelling types [10], suggesting that P/R signatures are indeed quite stable. In fact, cancer cell lines range from small, highly proliferative cells expressing the epithelial marker E-cadherin but not the mesenchymal marker vimentin, to another extreme of large, mesenchymal cells expressing vimentin but not E-cadherin. These two groups of cell lines respond differently to anticancer drugs [10]. As expected, the highly proliferative cell lines are more sensitive to antifolates and other antimetabolites. On the other hand, the mesenchymal cells are more sensitive to treatment with cholesterol synthesis inhibitors (statins) and mammalian target of rapamycin (mTOR) inhibitors. This is a surprising observation. One would expect that the highly proliferative cells would be more dependent on most biosynthetic pathways. While this is indeed the case for one-carbon metabolism (targeted by antifolates) and nucleotide metabolism (targeted by antimetabolites), it seems to be the opposite for protein synthesis (targeted by mTOR inhibitors) and cholesterol synthesis (targeted by statins). Mesenchymal cells are actually dependent on de novo cholesterol synthesis while epithelial cells can scavenge cholesterol from the media [11]. E-cadherin expression to the membrane is required for resistance to statin treatment, but it is not clear whether this localization is required for cholesterol transport into the cells.

Gene expression analysis can further be utilized to interrogate the activity of specific metabolic pathways and relations between the activities of different pathways. Pathway analysis is based on the annotation of genes that are related to specific pathways and some measure of pathway activity based on the expression of genes annotated for each pathway. Most pathway annotation databases such as Gene Ontology [12] and Kyoto Encyclopedia of Genes and Genomes (KEGG) [13] contain categories associated with metabolism. Several measures of pathway activity based on gene expression can be used, going from simple quantities like mean or median expression to more sophisticated quantifications from median polish analysis (MPA) [14, 15] or Gene Set Enrichment Analysis (GSEA) [16]. The outcome is a quantification of the activity of each metabolic pathway considered on each sample analysed. A study in the context of one-carbon metabolism in cancer cells shows that pathway activity, as quantified from gene expression, is a good predictor of metabolic flux, as estimated from ^13^C tracing experiments [17]. Thus, pathway activity can be used as a surrogate of pathway metabolic flux.

The discussions above about metabolic signatures associated with tissue of origin, cell proliferation and tissue remodelling are examples of pathway analysis applications to investigate the differential utilization of metabolic pathways across cancer subtypes. There have been several other studies using metabolic pathway analysis to generate hypotheses about the use of specific metabolic pathways in specific contexts. Examples include the dependency of p53 tumours on the mevalonate pathway [18], the increased activity of mitochondrial serine, one-carbon and glycine metabolism in cancer [17, 19, 20] and the regulation of serine biosynthesis by p73 [21].

There are currently other high-throughput platforms besides gene expression microarrays to interrogate metabolism genome-wide. The development of next-generation sequencing technologies has lowered the costs of DNA sequencing (DNAseq), providing the means to investigate the patterns of DNA alterations genome-wide across several cancer samples. Next-generation sequencing can also be applied to interrogate the whole or subsets of expressed RNAs (RNAseq), and it is replacing microarrays for the quantification of RNA expression. DNAseq and RNAseq, together with microRNA (miRNA) and methylation arrays, have been deployed to characterize the samples collected by The Cancer Genome Atlas (TCGA) project as well as the International Genome Consortium (IGC) project. This provides a unique opportunity to investigate cancer metabolism across multiple cancer subtypes and multiple genomic platforms. The analysis of RNAseq data linked to the TCGA samples corroborates the gene expression microarray analysis: the expression of metabolism genes is primarily dominated by tissue of origin [22]. It is worth noticing that the dominance of the tissue of origin signals extends beyond metabolism-related genes and beyond gene expression. An unsupervised clustering analysis of the TCGA samples revealed that whether it is RNAseq gene expression quantification, DNA sequencing, DNA methylation or all profiles together, the samples cluster by tissue type [23]. At the same time, RNAseq provides more detailed and more accurate data that produces high-throughput information in better alignment with low-throughput experimental techniques such as PCR. Thus, it is much more suitable particularly for in-depth mechanistic analyses within tissues or tissue classes.

#### Current challenges

RNAseq is rapidly replacing gene expression arrays as the standard technique for gene expression profiling. RNAseq provides a better quantification of transcript abundance, and the technology can be tailored to interrogate specific RNA subsets such as miRNAs and long non-coding RNAs. On the other hand, current established methods for gene signature analysis were tailored for gene expression arrays. They assume that the expression distribution for each probe across samples is close to a normal distribution or at least symmetric. RNAseq data violates these assumptions and may require new methodologies to conduct pathway analysis. The capability of RNASeq to resolve the expression of different gene isoforms together with the fact that different isoforms may have different enzymatic activities indicates that further developments are also required from the point of view of gene annotation. This could imply changes to commonly used databases like Gene Ontology, KEGG and other pathway annotation systems.

Most published cancer gene expression analyses focus on a snapshot and cell population average sampling of cancer tissue, potentially missing the dynamics and spatial heterogeneity of metabolism. Single-cell expression analysis and collection of samples at multiple time points could overcome this limitation, albeit with a dramatic increase in cost and effort. Therefore, statistical models are needed to infer the expression patterns of mixed cell types in a cancer sample, their metabolic state and their stage of progression.

### Genome-scale flux balance models

**Fig. 2:**
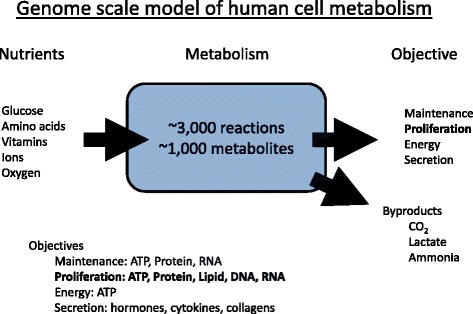
Core schema of genome-scale flux balance models. The construction of a core genome-scale flux balance model requires the specification of three major ingredients: the nutrients that are present in the extracellular media, the set of biochemical reactions that are encoded by the genome of the cells under study and the cell metabolic objective

The metabolic pathway analyses described above are based on pathway annotations and gene expression alone. Further elements are required to move from qualitative predictions (active/inactive) to quantitative predictions of the metabolic pathway rates. In principle, the pathway annotations, combined with a quantification of the cell biomass composition and metabolic objectives, could be used to determine which metabolic pathways should be active and at what rate, under specified culture conditions [24] (Fig. 2). Any attempt to model the system dynamics will require kinetic parameters characterizing the kinetic models for each reaction. Given that kinetic parameters are not available for most reactions, we first focus on metabolism at a steady state. By a steady state, we mean that the concentration of metabolites and the rate of biochemical reactions remain constant in time. The metabolic models that are constructed under the steady-state assumption are often called flux balance models.

The steady-state rate of biochemical reactions (change in concentration per unit of time) is often called flux, although it is not a flux as defined in physics and chemistry (rate of flow per unit area). The collection of steady-state rates of all reactions is called flux vector. At a steady state, the rate of production and consumption of every metabolite balances (flux balance constraint). *Bound constraints* on individual metabolic fluxes can be applied whenever available. For example, exchange fluxes of nutrients between the culture media and cells can be estimated from changes in the media metabolite concentrations and the cell number, also known as *consumption and release* (CORE) *profiles* [25]. Using the flux balance and bound constraints, we can attempt to identify the metabolic flux distribution that satisfies the cell metabolic objective at the specified rate and make use of the specified nutrient composition of the extracellular media. However, because cell metabolism is highly redundant, there are several flux distributions satisfying the typical metabolic objectives of mammalian cells (e.g. energy, proliferation) for the typical composition of the extracellular media (glucose, amino acids, etc.).

From an evolutionary point of view, we hypothesize that redundancy evolved to “efficiently” cope with different environmental conditions and constraints acting on cell metabolism. For example, cells with a metabolic flux vector that minimizes nutrient consumption while achieving a specified metabolic objective will be able to carry on that metabolic objective for longer times. In the context where only one nutrient is limiting the metabolic rate, minimizing the nutrient uptake given the metabolic objective rate is equivalent to maximizing the metabolic objective rate given the nutrient uptake rate. In one of the earlier applications of flux balance modelling to study mammalian cell growth, Savinell and Palsson investigated the flux distributions that satisfied a specified growth demand while minimizing the nutrient uptake [26, 27]. The minimization of nutrient uptake was implemented as a linear optimization objective with non-zero coefficients for every nutrient. They considered two scenarios of nutrient cost, molar cost where all the nutrient coefficients were set to 1 and mass cost where the nutrients cost were set to their molar masses. Some differences were noted regarding the differential utilization of glucose and amino acids depending on whether the molar or mass cost was applied. In either case, the energy requirements were satisfied by OxPhos in the mitochondria [26]. It is well known that for most nutrients, energy can be generated only through OxPhos. Although glucose has anaerobic fermentation to lactate as a second alternative, OxPhos has a higher yield of ATP per molecule of glucose than glycolysis to lactate. Therefore, the prediction of OxPhos as the main pathway for energy generation under aerobic conditions is in agreement with our intuition of efficiency per unit of nutrient.

However, most cancer cells generate a significant amount of energy from the metabolism of glycolysis to lactate even when growing in aerobic conditions (aerobic glycolysis, Warburg effect [28]). The failure to recapitulate the Warburg effect using flux balance and minimization of nutrient uptake alone indicates that a key ingredient is missing. It has been observed that the respiration rate remains approximately constant at high proliferation rates in spite of the increased energy requirements of biosynthesis [29]. This observation can be translated to the model adding a constraint to the oxygen consumption rate. As expected, when an upper bound is imposed in the oxygen consumption rate, aerobic glycolysis is predicted to become active when the oxygen consumption of OxPhos exceeds the imposed threshold (in *E. coli* [30] and unpublished data for mammalian cells).

The observed saturation in the oxygen consumption rate could be due to a limitation in the oxygen supply or a limitation in the oxidative phosphorylation capacity. Work in the context of muscle cell metabolism has shown that a further increase in the oxygen supply during aerobic conditions does not alter the respiration and aerobic glycolysis rates [31, 32], ruling out a limitation in the oxygen supply. Regarding the other alternative, a limitation in the oxidative phosphorylation capacity, cells could in principle increase their mitochondrial content to satisfy their higher energy demands. The crucial point is however that there is a limit on mitochondrial content.

The cell volume is crowded with cytoskeletal filaments, ribosomes, metabolic enzymes and organelles. Overexpression of any component is only possible at the expense of degradation of others. This macromolecular allocation constraint is analogous to the concept of solvent capacity in chemistry, reflecting the limited amount of solute that can be dissolved in a solvent. It was originally introduced under the name of molecular crowding [33] or solvent capacity constraint [34, 35]. We would like to introduce the name *macromolecular capacity constraint* because it reflects the limited amount of macromolecules that can be allocated in the cell volume.

The impact of the macromolecular capacity constraint is determined by the size of the macromolecules of interest. In this context, metabolic efficiency aims to minimize the impact of molecular crowding or, equivalently, to maximize metabolic rate per unit of volume occupied by the metabolic machinery. Based on data for in vitro reconstituted glycolysis at 30 °C, aerobic glycolysis can produce 0.73 mol ATP/min/(liters of glycolysis enzymes), calculated as 0.58 mmol lactate/min/(grams of glycolysis enzymes) [36] divided by a protein specific volume of 0.79 mL/g [37]. For cancer cell mitochondria, we obtain values equal or below 0.042–0.049 mol ATP/min/(liters of mitochondria), calculated as 0.11–0.13 mmol ATP/min/(grams of mitochondria protein) [38, 39], divided by a mitochondria specific volume of 2.63 mL/(grams of mitochondria protein) [40]. In this sense, aerobic glycolysis is 10 times more efficient than cancer cell mitochondria.

A mathematical model of energy metabolism based on flux balance, the minimization of nutrient utilization together with the macromolecular capacity constraint, is sufficient to explain the Warburg effect [41]. At low energy demands, the macromolecular capacity is irrelevant and energy is generated from OxPhos, the pathway with the highest yield of ATP per molecule of glucose. In contrast, at high energy demands, when the required mitochondrial content would exceed the macromolecular capacity, aerobic glycolysis must become active, producing ATP with a low requirement of intracellular space at expenses of a low yield of ATP per molecule of glucose.

**Fig. 3:**
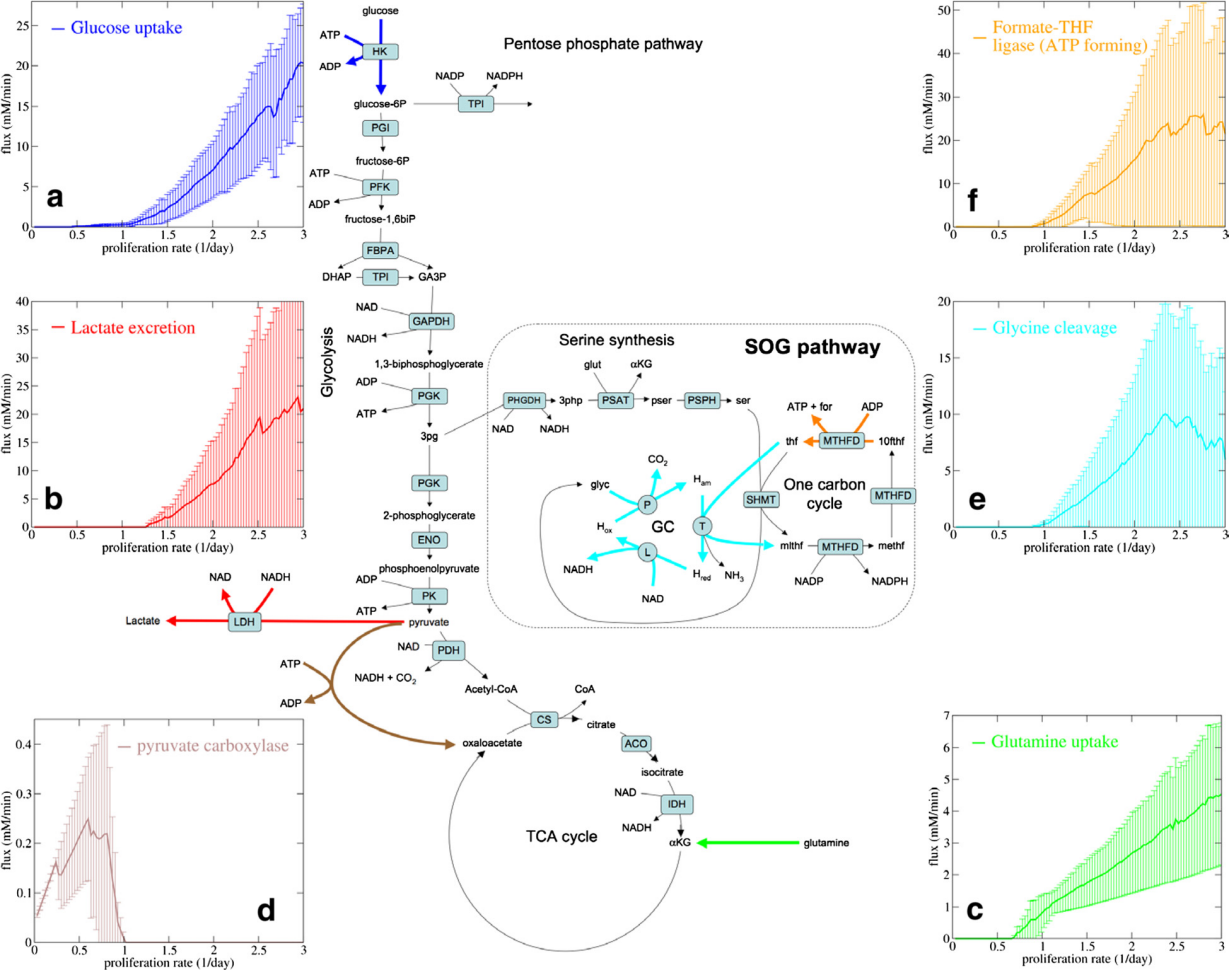
Predicted metabolic switch. Steady-state metabolic fluxes as a function of the proliferation rate, as predicted from simulations of a genome-scale flux balance model with the macromolecular capacity constraint [45]. The model contains kinetic parameters that are unknown and were sampled from a specified distribution. The *line* represents the median behaviour and the *error bars* the 90 % confidence intervals. **a**) Glucose uptake. **b**) Lactate excretion. **c**) Glutamine uptake. **d**) Pyruvate carboxylase. **e**) Glycinine cleavage. **f**) FTHFL. Sum of the reverse FTHFL flux of the cytosolic and mitochondrial enzymes

The flux balance modelling approach described above has been applied to genome-scale reconstructions of the human metabolic network. Most of the work has been based on the human metabolic network reconstruction from the Palsson group (Recon 2, [42]), although alternative reconstructions have been reported [43]. Simulations of genome-scale flux balance models of human metabolism demonstrate that the macromolecular capacity constraint implies metabolic changes beyond energy metabolism [44–46] (Fig. 3). In addition to increased glucose consumption (Fig. 3a), the genome-scale models predict the activation of glutamine uptake as cells increase their proliferation rate [44, 45] (Fig. 3c). At low proliferation rates, when glutamine uptake is predicted inactive, pyruvate carboxylase is predicted to satisfy the anaplerotic requirements of the TCA cycle [46] (Fig. 3d). This prediction agrees with the requirement of pyruvate carboxylase in glutamine-free media [47]. These additional metabolic changes are a consequence of another feature of synthesis of biomass precursors (e.g. amino acids, AcCoA) from glucose: NADH production [48]. The biosynthesis of biomass precursors from glucose involve some NAD^+^-dependent dehydrogenases resulting in a net production of NADH. The generated NADH can be used via OxPhos to generate energy. However, once again, when the OxPhos capacity is exceeded, cells should find other means to synthesize precursor metabolites without NADH generation. This can be achieved by importing non-essential amino acids from the media and by synthesizing AcCoA for alternative sources. Among amino acids, only glutamine, glutamate, phenylalanine and tyrosine can be used to produce AcCoA without NADH generation [48].

The genome-scale models also predict an increase in the rate of serine, one-carbon and glycine (SOG) metabolism [45] (Fig. 3f, e). The rate of the SOG pathway is predicted to further increase when the pyruvate kinase reaction is removed from the model or when the pyruvate kinase activity is uncoupled from ATP production [45]. That prompted us to postulate the SOG pathway as a novel pathway for ATP generation. The ATP generation step is given by the reverse activity of 10-formyl-tetrahydrofolate synthase (FTHFL, Fig. 3f). ATP production by FTHFL is supported by kinetic analysis of *C. cylindrosporum* FTHFL [49] and by kinetic modelling of mammalian FTHFL [45]. Treatment of cancer cells with the antifolate methotrexate induces an energy stress, providing indirect evidence of energy production by folate metabolism [20]. However, further experimental evidence is required to ascertain the contribution of SOG pathway to ATP generation in mammalian cells. The SOG pathway also contains dehydrogenase steps that could contribute to NADPH production (Fig. 3f). The importance of this observation was not recognized until recently [20, 50]. It has been experimentally validated that NADP^+^ dehydrogenases from one-carbon metabolism contribute to NADPH generation in the cytosol and the mitochondria [50].

Flux balance models can be further constrained to take into account metabolic enzyme expression patterns that are specific to a given tissue or that are the consequence of molecular alterations present in cancer cells. For example, a given cancer may have a homozygous deletion of a genomic region containing one or more genes coding for metabolic enzymes. A more accurate metabolic model of these cancer cells should have the corresponding biochemical reactions removed. Furthermore, as shown above, the analysis of gene expression patterns across cancers and normal tissues reveals that the metabolism of cancer cells closely resembles the metabolism of the tissue of origin [9]. These molecular alterations and tissue of origin biases may have a significant impact on the cancer cell metabolism.

A recent community-driven effort has combined the annotation of the human metabolic network together with protein expression data to obtain 65 cell-type-specific metabolic models (Recon X, [24]). Several methodologies have been developed to tailor a reconstruction of the human metabolic network to a specific cell type (*personalized* model), using as input expression profiles, proteomics or other genomic data [51–55]. Personalized flux balance models have been tailored to investigate the metabolism of cancer cells with specified alterations. In the simplest case scenario, one can model metabolism in the context of inactivation of one or more enzymes, simply removing the corresponding reaction from the model. This approach led to the identification of heme oxygenase as an essential reaction in cancers with fumarate hydratase deficiency [56]. Moving to a genome-wide approach, gene expression profiles can be used to personalize generic genome-scale flux balance models and obtain a more accurate representation [57, 58]. These personalized flux balance models find flux distributions that satisfy the constraints of generic flux balance models (as described above) and that are more consistent with the expression patterns of genes coding for the enzymes catalysing the corresponding reactions. Personalized metabolic flux balance models have been used to investigate tissue-specific metabolism [57], to predict cell line-specific metabolic vulnerabilities [58] and to identify putative oncometabolites [59].

#### Current challenges

It is becoming evident that a realistic flux balance model of cell metabolism should incorporate the macromolecular capacity constraint. However, a precise implementation of the molecular capacity constraint requires reliable estimates of kinetic parameters. Current implementations sample the kinetic parameters from a specified distribution [30], reporting typical fluxes and confidence intervals (see for instance Fig. 3). However, there are intracellular pathways with a high degree of redundancy, where the model predictive power is dramatically reduced. For example, the complementarity between cytosolic and mitochondrial folate metabolism results into confidence intervals as wide as the average flux values for folate metabolism reactions [20]. This redundancy can be also linked to the existence of alternative pathway for the formation of an end product. For example, aerobic glycolysis and the putative SOG pathway can both generate ATP, resulting in wide confidence for the lactate by-product of aerobic glycolysis (Fig. 3b) and the FTHFL reverse flux (Fig. 3f).

Personalized flux balance models can be also limited by the lack of relevant data to constraint the model. In the path from gene expression, protein expression and enzyme activity to reaction rate, there are regulatory points. Post-transcriptional regulation may result in the lack of proportionality between gene expression and reaction rate. This caveat is in part corrected by the fact that the flux balance model searches for solutions with the best consensus agreement between all reaction rates and the expression of genes coding for the corresponding enzymes. In fact, inconsistency between gene expression and predicted reaction flux can be used to infer reaction steps where post-translational regulation may be taking place [57]. Future work should address this point in further detail by using as input proteomics and phospho-proteomic data [60].

The application of personalized flux balance models to cancer samples extracted from animal models or patients is subject to the additional caveat of cells with mixed metabolic phenotypes. The gene expression profiles, or proteomic profiles, represent an average over all cells present in the extracted sample. This average expression is informative only if most cells exhibit a dominant expression pattern. However, in the context of two or more population of cells in significant proportions and metabolic differences, any prediction based on population averages can be misleading. To tackle this scenario, we need mathematical methodologies to infer the different cell subtypes and disentangle their expression profiles or to deploy experimental protocols to separate and profile the different cell populations.

### Genome-scale flux balance models with kinetics

Whenever available, the kinetic model of biochemical reactions and estimates of the corresponding kinetic parameters can significantly improve model predictions. Kinetic models have been used extensively in the past to investigate selected metabolic pathways. Some examples are highlighted in Table 2. The next step is to bring a kinetic description to genome-scale models. This problem can be divided in two major challenges: kinetic annotation and model solution.

**Table 2:** Kinetic models. Selected kinetic models of metabolic pathways, focusing on reaction kinetics and parameters for mammalian cells

Pathway	Tissue	Major conclusion	Reference
Glycolysis	Cancer cells	Glycolysis has different control steps depending on cell line	[88]
Glycolysis	Cancer cells	GAPDH is the rate limiting step of glycolysis	[89]
Pentose phosphate pathway	Hepatocytes	G6P dehydrogenase controls the oxidative rate and transketolase the non-oxidative rate	[90]
One-carbon metabolism	Hepatocytes	Mitochondrial formate is the major source of cytosolic one-carbon units in proliferating hepatocytes	[91]
H_2_O_2_ elimination	Endothelial cells	GSSG reductase controls the NAPDH dependent H_2_O_2_ elimination	[92]
Mitochondria	Hepatocytes	The rate of superoxide generation is a function of the proton electrochemical potential	[93]
Central metabolism	Cancer cells	Repression of transaldolase and succinyl-CoA ligase and the synergistic combination of transaldolase and serine hydromethyltransferase significantly reduce growth rate	[94]

For the kinetic annotation, we can start compiling all reported kinetic models and parameters in studies like those highlighted in Table 1. For reactions with no annotations, we can deploy generic kinetic models. *Generic kinetic models* of biochemical reactions aim to capture the key features of enzyme kinetics [61–65]. The key factors are the enzyme turnover rate, the enzyme concentration, a substrate saturation term and a thermodynamics term associated with the enzyme properties at equilibrium. Even these generic kinetic models contain kinetic parameters that must be estimated. To address that problem, we first need to discuss how to solve genome-scale kinetic models.

Flux balance models can be generalized to include kinetic data and improve the estimation of steady-state metabolic fluxes. As before, a metabolic objective (e.g. proliferation), metabolic constraints (e.g. available nutrients, macromolecular capacity) and an efficiency principle (e.g. minimize nutrient uptake) are specified. But now we take into account that the amount of enzyme needed to maintain a specified reaction rate depends on the concentration of substrates and products via the corresponding kinetic model. With this, the optimization problem searches not only for the optimal steady-state fluxes but also for the optimal metabolite concentrations.

Flux balance models with kinetics have been applied to the study of yeast [35] and *E. coli* [66, 67] glycolysis. In these studies, the kinetic models and parameters for every reaction were specified and the optimal metabolite concentrations were determined. In both yeast and *E. coli*, there was a good agreement between the predicted optimal metabolite concentrations and the typical reported values. The optimal metabolite concentrations depend to a great extent on the reactions equilibrium constant.

#### Current challenges

The extension of flux balance models with kinetics to genome scale is on its way. The major challenge is the estimation of missing kinetic parameters or other information. Workflows to address this problem have been reported and applied to genome-scale models of different organisms [65, 68, 69]. An iterative reconstruction approach has been also proposed for the reconstruction of the metabolic network, reaction kinetic laws and kinetic parameters [70]. Therefore, it seems just a matter of time for the deployment of those methods to develop a genome-scale flux balance model with kinetics of a human cell. In the meantime, an interesting mathematical result demonstrates that we are walking on solid ground. Thanks to the macromolecular capacity constraint, the optimal metabolic flux distributions are elementary flux modes satisfying the metabolic objective [71, 72]. *Elementary flux modes* were defined as minimal metabolic flux distributions that are both stoichiometrically and thermodynamically feasible [73]. Therefore, the molecular capacity constraint forces cell metabolism into elementary flux modes. Whether this represented a selective advantage for the evolution of molecular crowding is an open question.

### Reaction diffusion models

Some metabolites may exhibit significant concentration gradients within cellular compartments. Accounting for those concentration gradients can increase the level of realism of metabolic models, albeit with increased model complexity. To model metabolism in this context, we need to resort to reaction diffusion models, characterizing the spatio-temporal variations of the metabolite concentrations coupled to the reaction dynamics consuming/producing metabolites.

It has been recently proposed that glycolytic enzymes and mitochondria are located in different cell regions to satisfy different energetic demands [74]. In this model, mitochondria localize to the peri-nuclear area where OxPhos efficiently supplies the sustained energy demand of biosynthesis, while glycolysis is necessary to supply rapid energy demands primarily to support membrane pumps. The mathematical description of this scenario requires reaction diffusion equations accounting for the existence of gradients from the cell membrane to the peri-nuclear area [74]. Although this model may sound appealing, the reported experimental evidence is not sufficient to prove its validity. Increased glycolysis following overexpression of a cell membrane ATPase was taken as evidence that the role of glycolysis is to supply rapid energy demand at the cell membrane. However, what “rapid” means is not clear since aerobic glycolysis remained high after chronic overexpression of the cell membrane ATPase. More importantly, a control experiment where an ATPase is overexpressed at some other cell compartment was not provided.

#### Current challenges

The point that subcellular localization of the molecular machinery producing/consuming energy may result in concentration gradients should be taken into consideration. Membrane pumps are indeed major sites of energy consumption during osmotic stress as hypothesized above [74]. During osmotic stress, there are additional energy requirements associated with cytoskeleton remodelling and biosynthesis of metabolites involved in cell volume regulation [75]. Patterns of subcellular localization have been also observed during cell migration. Mitochondria localize at the leading edge of migrating cancer cells and a causal relation between the degree of that localization and the migration speed has been demonstrated [76]. Finally, different hexokinase isoenzymes localize to the cytosol or the mitochondrial membrane in a context-dependent manner [77]. Future work should focus on the development of reaction diffusion models aiming to understand the consequences of this subcellular localization patterns.

### Tumour microenvironment models

Tumour microenvironment models aim to understand how properties of cancer and tumour stroma cells and their cellular interactions determine macroscopic parameters such as tumour growth rate. The remodelling of the tumour microenvironment requires cancer and stroma cells to acquire metabolic capabilities beyond cell proliferation. Furthermore, cancer cells located in different tumour regions may experience different microenvironments, and they may require different metabolic strategies to survive, proliferate and disseminate. In the following, we describe different approaches to model tumours with an emphasis on tumour metabolism.

#### Reaction diffusion models

In reaction diffusion models, tumour and stroma cells are represented by spatio-temporal distributions (fields) quantifying the density of normal and cancer cells. The evolution in time and space of these distributions is described by partial differential equations accounting for relevant processes and constraints. The processes/constraints most commonly considered are *cell proliferation*, characterizing the cell population growth, and *cell motility*, characterizing the migratory movements depending on limited resources. Additional partial differential equations can be added to model different aspects of metabolism, including the spatio-temporal variations of nutrients and excreted by-products.

Using a reaction diffusion model with one type of stroma cell, one type of cancer cell and a spatio-temporal distribution of tumour-secreted protons (a surrogate of acidification), Gatenby et al. were able to reproduce characteristic features of tumours [78, 79], including tumour wave front velocity, pH gradient and crossover from localized to invasive tumour. This work emphasizes the relevance of acidification due to the Warburg effect in tumour development.

#### Soft matter models of tumours

The properties of tumours can be investigated focusing on their mechanical behaviour, building on soft matter models of developing tissues [80]. The major challenge here is to identify relationships between properties characterizing cells and cell-cell interactions to the tumour mechanical properties [81, 82]. Using soft matter models, it has been shown that tumours behave like viscoelastic fluid [83]. Following quick small perturbations, the tumour reacts as an elastic material but it behaves like liquid for steady and/or large stresses. Interestingly, soft matter models predict that the fluidity of the tumour increases with increasing rate of cell proliferation [83], connecting a mechanical property (tumour fluidity) with a metabolic one (cell proliferation).

Homeostatic pressure is an interesting concept emerging from soft matter models [84]. The *homeostatic pressure* is defined as the pressure that needs to be applied to keep the tumour volume constant. If the applied pressure is smaller than the homeostatic pressure, then the tumour will continue growing; if it is larger, then the tumour will start shrinking. The homeostatic pressure may have some advantage over tumour growth rate as a measure of tumour malignancy. The tumour growth rate is determined by the difference between the tumour homeostatic pressure and the pressure being applied by the surrounding tissue. In that sense, the tumour growth rate is not an intrinsic property of the tumour; it also depends on its surrounding. In contrast, the homeostatic pressure is an intrinsic tumour property. However, there is currently no protocol to measure homeostatic pressure in vivo.

#### Current challenges

Future work is required to understand how cancer metabolism influences the mechanical properties of tumours. This relation will be in part dictated by the influence of metabolism on cell proliferation and the relation between cell proliferation and the tumour fluidity described above. In addition, it is well documented that metabolic by-products, most notably lactate and protons, play a role in the remodelling of the tumour microenvironment. The translation of these relationships to models and laws will allow us to use tumour mechanical properties as indirect surrogates to investigate cancer metabolism in vivo, using non-invasive techniques such as ultrasound.

When speaking about the tumour environment, we tend to focus on the tissue that is in close spatial proximity to the cancer cells (microenvironment). However, we should not forget that the blood circulation system connects the metabolism in the cancer tissue with the organism metabolism (macroenvironment). In other words, a complete mathematical description of the tumour microenvironment should take into account the interaction between cancer cells and distant organs via the circulatory system and, by extrapolation, with nutrition. The manifestation of the muscle-wasting syndrome of cachexia in cancer patients is a demonstration of these distant interactions [85]. Therefore, a definitive model of cancer metabolism should account for the interactions between cancer cells and distant organs. From the technical point of view, this will require to link metabolic models for the cancer cells, the stroma cells and the relevant distant organs such as the liver. The good news is that the community efforts in the reconstruction of the human metabolic network have already provided the first drafts of tissue-specific metabolic models [24], and some advanced refinements are already available for liver metabolism [86].

## Conclusions

We have reviewed five major areas in mathematical models of metabolism, each addressing specific questions at different scales. First, pathway expression analysis is probably the best means to understand the heterogeneity of metabolism across cancers and to create hypotheses about major cancer metabolism subtypes. Second, flux balance models are a flexible framework to investigate the selective advantage of different metabolic pathways depending on the environmental conditions and the metabolic objective. Gene expression and other genomic information can be used to tailor flux balance models to specific cancer metabolic subtypes, allowing us to make predictions of their specific metabolic vulnerabilities. Third, kinetic modelling is required to obtain an accurate model of cell metabolism, and it is essential to understand the relationships between metabolic fluxes and metabolite concentrations. Fourth, there is experimental evidence of patterns of spatial localization of energy metabolism-related enzyme and mitochondria. The relevance of this spatial heterogeneity can only be addressed with reaction diffusion models of cell metabolism. Finally, tumour microenvironment models will help us to understand how metabolic alterations can impact the remodelling of the tumour tissue.

Pathway expression analysis indicates that the tissue of origin is the first dominant signature in the genome-wide and metabolism-specific gene expression profiles of cancers. After correcting for the tissue of origin, cell proliferation and tissue remodelling emerge as secondary major signatures. Gene signatures associated with key metabolic pathways such as glycolysis, the pentose phosphate pathway, oxidative phosphorylation and one-carbon metabolism are correlated to a great extent with the degree of proliferation. In contrast, a gene signature representing autophagy manifests the strongest expression in cancers with a high degree of tissue remodelling. Other parts of the metabolic system, such as fatty acid metabolism, are not generally associated with these major factors. This could be due to high levels of redundancy and feedback obscuring the pathway analysis; however, there could also be other factors associated with the utilization of these metabolic regimes. More detailed studies within individual tissues and tissue classes are needed to gain a better understanding of the variation and dependencies of metabolic pathways.

The evolution of flux balance models is getting closer to the development of genome-scale flux balance models with kinetics. It is an exciting time in this area. The increase in model complexity and burden in parameter estimation is balanced by a significant reduction of the space of possible flux distributions to elementary flux modes. The next step is to develop methods to estimate large sets of kinetic parameters from proteomic, phospho-proteomic and metabolomic profiles. Once these methods have been applied, we will be in a better position to address open questions regarding the selective advantage of metabolic phenotypes of cancer cells.

The investigation of tumour metabolism using tumour microenvironment models is in its early days, as it is the case in the experimental field as well. However, these models are required to understand the selective advantage of metabolic interactions between cancer cells and the stroma. There are reports of both glycolytic cancer cells feeding lactate to the stroma and glycolytic stroma feeding lactate to the cancer cells [87]. It remains to be elucidated in which context these opposite metabolic phenotypes are selected for.

## Abbreviations

CORE: consumption and release
DNAseq: DNA sequencing
FTHFL: 10-formyl-tetrahydrofolate synthase
GEO: Gene Expression Omnibus
IGC: International Genome Consortium
OxPhos: oxidative phosphorylation
P: proliferation
R: remodelling
RNAseq: RNA sequencing
SOG: serine, one-carbon and glycine metabolism
TCGA: The Cancer Genome Atlas
